# 1,4-Bis(4-pyridylsulfanylmeth­yl)benzene

**DOI:** 10.1107/S1600536808017571

**Published:** 2008-06-19

**Authors:** Suk-Hee Moon, Ki-Min Park

**Affiliations:** a Subdivision of Food Science, Kyungnam College of Information and Technology, Busan 616-701, Republic of Korea; b Research Institute of Natural Science, Gyeongsang National University, Jinju 660-701, Republic of Korea

## Abstract

In the title compound, C_18_H_16_N_2_S_2_, a crystallographic inversion centre lies at the centre of the benzene ring, and the two terminal 4-mercaptopyridyl groups adopt an *anti* geometry. Each benzene ring makes a dihedral angle of 55.4 (1)° with the plane of the benzene fragment. The crystal structure is stabilized by C—H⋯π inter­actions between a benzene H atom and a pyridyl ring of a neighbouring mol­ecule. In addition, the crystal structure exhibits inter­molecular C—H⋯N inter­actions.

## Related literature

For details of the preparation and related structures of 1,4-bis­(2-pyridyl-sulfanylmeth­yl)benezene derivatives, see: Atherton *et al.* (1999 ▶); McMorran & Steel (2003 ▶); For the structures of Co(II) and Ag (I) complexes of 1,4-bis­(2-pyridylsulfanylmeth­yl)benezene, see: Hartshorn & Steel (1998 ▶). For bond-length data, see: Allen *et al.* (1987 ▶).
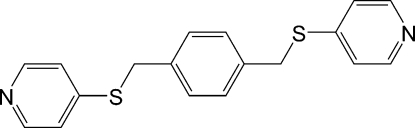


## Experimental

### 

#### Crystal data

C_18_H_16_N_2_S_2_
*M* *_r_* = 324.45
Monoclinic, 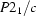

*a* = 7.145 (1) Å
*b* = 6.1667 (8) Å
*c* = 17.954 (2) Å
β = 90.391 (3)°
*V* = 791.03 (18) Å^3^
*Z* = 2
Mo *K*α radiation
μ = 0.33 mm^−1^
*T* = 298 (2) K
0.35 × 0.20 × 0.15 mm

#### Data collection

Bruker SMART CCD area-detector diffractometer
Absorption correction: none
4706 measured reflections
1717 independent reflections
893 reflections with *I* > 2σ(*I*)
*R*_int_ = 0.074

#### Refinement

*R*[*F* ^2^ > 2σ(*F* ^2^)] = 0.052
*wR*(*F* ^2^) = 0.121
*S* = 0.96
1717 reflections
100 parameters
H-atom parameters constrained
Δρ_max_ = 0.32 e Å^−3^
Δρ_min_ = −0.17 e Å^−3^

### 

Data collection: *SMART* (Bruker, 2000 ▶); cell refinement: *SAINT-Plus* (Bruker, 2000 ▶); data reduction: *SAINT-Plus*; program(s) used to solve structure: *SHELXTL* (Sheldrick, 2008 ▶); program(s) used to refine structure: *SHELXTL*; molecular graphics: *SHELXTL* and *DIAMOND* (Brandenburg, 1998 ▶); software used to prepare material for publication: *SHELXTL*.

## Supplementary Material

Crystal structure: contains datablocks I, global. DOI: 10.1107/S1600536808017571/lx2058sup1.cif

Structure factors: contains datablocks I. DOI: 10.1107/S1600536808017571/lx2058Isup2.hkl

Additional supplementary materials:  crystallographic information; 3D view; checkCIF report

## Figures and Tables

**Table 1:** Hydrogen-bond geometry (Å, °) *Cg* is the centroid of N1/C1/C2/C3/C5 pyridyl ring.

*D*—H⋯*A*	*D*—H	H⋯*A*	*D*⋯*A*	*D*—H⋯*A*
C1—H1⋯N1^i^	0.93	2.61	3.484 (4)	158
C8—H8⋯*Cg*^ii^	0.93	2.77	3.560 (4)	143

Symmetry codes: (i) 
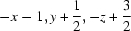
; (ii) 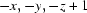.

**Fig. 1.**
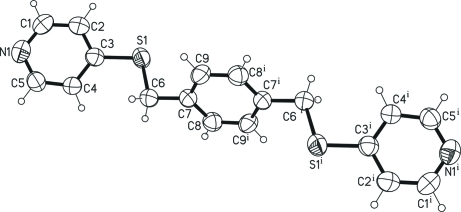
The molecular structure of (I), showing the atom-numbering scheme. Displacement ellipsoids are drawn at the 50% probability level for non-H atoms. [Symmetry code: (i) -x + 1, -y + 1, -z + 1]

**Fig. 2.**
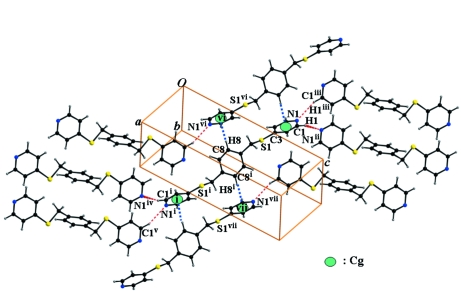
C—H···π and C—H···N interactions (dotted lines) in the title compound. Cg denotes the ring centroid. [Symmetry codes: (i) -x+1, -y+1, -z+1; (ii) -x-1, y+1/2, -z+3/2; (iii) -x-1, y-1/2, -z+3/2; (iv) x+2, -y+1/2, z-1/2; (v) x+2, -y+3/2, z-1/2; (vi) -x, -y, -z+1;(vii) x+1, y+1, z.]

## supplementary crystallographic information

### Comment

The reaction of α,α'-dibromo-*p*-xylene with 4-mercaptopyridine afforded the title compound, in which the crystallographic inversion centre lies on the centre of the benzene ring. Therefore, the asymmetric unit consists of a half of molecule and the two 4-mercaptopyridyl groups adopt an anti-geometry (Fig. 1). All bond lengths and angles show normal value (Allen *et al.*, 1987). The dihedral angle between the plane of benzene and the terminal pyridyl ring is 55.4 (1)°, which is smaller than those of related structures (Atherton *et al.*, 1999; Hartshorn & Steel, 1998).

The crystal packing (Fig. 2) is stabilized by C—H···π interactions between a benzene H atom and the pyridyl ring of neighbouring molecule, with a C8—H8···*Cg* separation of 2.77 Å (Fig. 2 and Table 1; *Cg* is the centroid of N1/C1/C2/C3/C5 pyridyl ring, symmetry code as in Fig. 2). The molecular packing (Fig. 2) is further stabilized by intermolecular C—H···N hydrogen bonds between a pyridyl H atom and the pyridine N atom of neighbouring molecule, with a C1—H1···N1^i^ separation of 2.61 Å (Fig. 2 and Table 1; symmetry code as in Fig. 2).

### Experimental

The title compound was prepared by the reaction of α,α'-dibromo-*p*-xylene with 4-mercaptopyridine in acetonitrile according to reported methods (Atherton *et al.*, 1999; McMorran & Steel, 2003). Single crystal suitable for X-ray analysis were obtained by evaporation of a solution of the title compound in acetonitrile.

### Refinement

All H-atoms were positioned geometrically and refined using a riding model with d(C—H) = 0.93 Å, *U*_iso_ =1.2*U*_eq_(C) for aromatic and 0.97 Å, *U*_iso_ = 1.2*U*_eq_(C) for CH_2_ atoms.

### Crystal data

**Table:**

C_18_H_16_N_2_S_2_	*F*_000_ = 340
*M**_r_* = 324.45	*D*_x_ = 1.362 Mg m^−^^3^
Monoclinic, *P*2_1_/*c*	Mo *K*α radiation λ = 0.71073 Å
Hall symbol: -P 2ybc	Cell parameters from 4706 reflections
*a* = 7.145 (1) Å	θ = 2.3–27.0º
*b* = 6.1667 (8) Å	µ = 0.33 mm^−^^1^
*c* = 17.954 (2) Å	*T* = 298 (2) K
β = 90.391 (3)º	Plate, colourless
*V* = 791.03 (18) Å^3^	0.35 × 0.20 × 0.15 mm
*Z* = 2	

### Data collection

**Table:**

Bruker SMART CCD area-detector diffractometer	893 reflections with *I* > 2σ(*I*)
Radiation source: fine-focus sealed tube	*R*_int_ = 0.074
Monochromator: graphite	θ_max_ = 27.0º
*T* = 298(2) K	θ_min_ = 2.3º
φ and ω scans	*h* = −9→7
Absorption correction: none	*k* = −7→7
4706 measured reflections	*l* = −21→22
1717 independent reflections	

### Refinement

**Table:**

Refinement on *F*^2^	Secondary atom site location: difference Fourier map
Least-squares matrix: full	Hydrogen site location: inferred from neighbouring sites
*R*[*F*^2^ > 2σ(*F*^2^)] = 0.052	H-atom parameters constrained
*wR*(*F*^2^) = 0.121	*w* = 1/[σ^2^(*F*_o_^2^) + (0.0516*P*)^2^] where *P* = (*F*_o_^2^ + 2*F*_c_^2^)/3
*S* = 0.96	(Δ/σ)_max_ = 0.001
1717 reflections	Δρ_max_ = 0.32 e Å^−^^3^
100 parameters	Δρ_min_ = −0.17 e Å^−^^3^
Primary atom site location: structure-invariant direct methods	Extinction correction: none

### Special details

**Table:**

Geometry. All e.s.d.'s (except the e.s.d. in the dihedral angle between two l.s. planes) are estimated using the full covariance matrix. The cell e.s.d.'s are taken into account individually in the estimation of e.s.d.'s in distances, angles and torsion angles; correlations between e.s.d.'s in cell parameters are only used when they are defined by crystal symmetry. An approximate (isotropic) treatment of cell e.s.d.'s is used for estimating e.s.d.'s involving l.s. planes.
Refinement. Refinement of *F*^2^ against ALL reflections. The weighted *R*-factor *wR* and goodness of fit *S* are based on *F*^2^, conventional *R*-factors *R* are based on *F*, with *F* set to zero for negative *F*^2^. The threshold expression of *F*^2^ > σ(*F*^2^) is used only for calculating *R*-factors(gt) *etc*. and is not relevant to the choice of reflections for refinement. *R*-factors based on *F*^2^ are statistically about twice as large as those based on *F*, and *R*- factors based on ALL data will be even larger.

### Fractional atomic coordinates and isotropic or equivalent isotropic displacement parameters (Å^2^)

**Table:**

	*x*	*y*	*z*	*U*_iso_*/*U*_eq_	
S1	0.01648 (12)	0.27398 (12)	0.56971 (5)	0.0651 (3)	
N1	−0.2740 (4)	−0.1917 (5)	0.72963 (13)	0.0677 (8)	
C1	−0.3414 (4)	0.0057 (6)	0.71296 (17)	0.0632 (9)	
H1	−0.4533	0.0486	0.7346	0.076*	
C2	−0.2540 (4)	0.1478 (5)	0.66562 (16)	0.0587 (8)	
H2	−0.3052	0.2842	0.6570	0.070*	
C3	−0.0897 (4)	0.0876 (4)	0.63081 (15)	0.0501 (7)	
C4	−0.0215 (4)	−0.1175 (5)	0.64684 (15)	0.0561 (8)	
H4	0.0875	−0.1672	0.6245	0.067*	
C5	−0.1161 (5)	−0.2463 (5)	0.69596 (15)	0.0593 (8)	
H5	−0.0660	−0.3822	0.7065	0.071*	
C6	0.2555 (4)	0.1735 (4)	0.56468 (16)	0.0620 (9)	
H6A	0.3003	0.1373	0.6143	0.074*	
H6B	0.2588	0.0430	0.5345	0.074*	
C7	0.3805 (4)	0.3443 (4)	0.53083 (15)	0.0493 (7)	
C8	0.4517 (4)	0.3178 (4)	0.46027 (16)	0.0537 (8)	
H8	0.4190	0.1961	0.4325	0.064*	
C9	0.4290 (4)	0.5300 (5)	0.56966 (15)	0.0554 (8)	
H9	0.3801	0.5526	0.6169	0.066*	

### Atomic displacement parameters (Å^2^)

**Table:**

	*U*^11^	*U*^22^	*U*^33^	*U*^12^	*U*^13^	*U*^23^
S1	0.0593 (5)	0.0556 (5)	0.0806 (6)	0.0050 (4)	0.0101 (4)	0.0205 (4)
N1	0.0699 (19)	0.0749 (19)	0.0584 (16)	−0.0087 (15)	0.0095 (14)	0.0062 (14)
C1	0.051 (2)	0.079 (2)	0.059 (2)	−0.0008 (18)	0.0091 (16)	−0.0047 (18)
C2	0.057 (2)	0.0542 (18)	0.065 (2)	0.0081 (16)	−0.0015 (16)	−0.0020 (17)
C3	0.0483 (19)	0.0475 (17)	0.0546 (17)	−0.0018 (14)	−0.0016 (14)	−0.0025 (14)
C4	0.059 (2)	0.0474 (17)	0.0616 (19)	0.0032 (15)	0.0121 (16)	−0.0005 (16)
C5	0.066 (2)	0.0527 (18)	0.0595 (19)	0.0022 (17)	−0.0004 (17)	0.0065 (15)
C6	0.056 (2)	0.0492 (17)	0.081 (2)	0.0074 (14)	0.0188 (17)	0.0112 (16)
C7	0.0475 (18)	0.0459 (17)	0.0546 (18)	0.0039 (13)	0.0049 (15)	0.0074 (14)
C8	0.0600 (19)	0.0493 (17)	0.0519 (18)	−0.0006 (15)	0.0007 (15)	−0.0040 (15)
C9	0.060 (2)	0.0618 (19)	0.0443 (17)	0.0053 (17)	0.0105 (15)	0.0009 (15)

### Geometric parameters (Å, °)

**Table:**

S1—C3	1.764 (3)	C5—H5	0.9300
S1—C6	1.820 (3)	C6—C7	1.511 (4)
N1—C5	1.327 (4)	C6—H6A	0.9700
N1—C1	1.342 (4)	C6—H6B	0.9700
C1—C2	1.374 (4)	C7—C8	1.378 (3)
C1—H1	0.9300	C7—C9	1.383 (4)
C2—C3	1.385 (4)	C8—C9^i^	1.379 (4)
C2—H2	0.9300	C8—H8	0.9300
C3—C4	1.385 (4)	C9—C8^i^	1.379 (4)
C4—C5	1.369 (4)	C9—H9	0.9300
C4—H4	0.9300		
			
C3—S1—C6	102.52 (13)	C4—C5—H5	117.6
C5—N1—C1	115.7 (3)	C7—C6—S1	109.87 (18)
N1—C1—C2	123.6 (3)	C7—C6—H6A	109.7
N1—C1—H1	118.2	S1—C6—H6A	109.7
C2—C1—H1	118.2	C7—C6—H6B	109.7
C1—C2—C3	119.9 (3)	S1—C6—H6B	109.7
C1—C2—H2	120.1	H6A—C6—H6B	108.2
C3—C2—H2	120.1	C8—C7—C9	117.9 (3)
C2—C3—C4	116.7 (3)	C8—C7—C6	120.7 (3)
C2—C3—S1	118.4 (2)	C9—C7—C6	121.4 (3)
C4—C3—S1	124.9 (2)	C7—C8—C9^i^	120.7 (3)
C5—C4—C3	119.3 (3)	C7—C8—H8	119.7
C5—C4—H4	120.4	C9^i^—C8—H8	119.7
C3—C4—H4	120.4	C8^i^—C9—C7	121.3 (3)
N1—C5—C4	124.8 (3)	C8^i^—C9—H9	119.3
N1—C5—H5	117.6	C7—C9—H9	119.3
			
C5—N1—C1—C2	−1.4 (4)	C3—C4—C5—N1	1.2 (5)
N1—C1—C2—C3	1.6 (5)	C3—S1—C6—C7	−165.4 (2)
C1—C2—C3—C4	−0.4 (4)	S1—C6—C7—C8	−109.1 (3)
C1—C2—C3—S1	179.4 (2)	S1—C6—C7—C9	71.7 (3)
C6—S1—C3—C2	160.6 (2)	C9—C7—C8—C9^i^	1.5 (5)
C6—S1—C3—C4	−19.7 (3)	C6—C7—C8—C9^i^	−177.7 (2)
C2—C3—C4—C5	−0.9 (4)	C8—C7—C9—C8^i^	−1.5 (5)
S1—C3—C4—C5	179.3 (2)	C6—C7—C9—C8^i^	177.7 (3)
C1—N1—C5—C4	−0.1 (5)		

Symmetry codes: (i) −*x*+1, −*y*+1, −*z*+1.

### Hydrogen-bond geometry (Å, °)

**Table:**

*D*—H···*A*	*D*—H	H···*A*	*D*···*A*	*D*—H···*A*
C1—H1···N1^ii^	0.93	2.61	3.484 (4)	158
C8—H8···Cg^iii^	0.93	2.77	3.560 (4)	143

Symmetry codes: (ii) −*x*−1, *y*+1/2, −*z*+3/2; (iii) −*x*, −*y*, −*z*+1.

## References

[bb1] Allen, F. H., Kennard, O., Watson, D. G., Brammer, L., Orpen, A. G. & Taylor, R. (1987). *J. Chem. Soc. Perkin Trans* *2*, pp. S1–19.

[bb2] Atherton, Z., Goodgame, D. M. L., Menzer, S. & Williams, D. J. (1999). *Polyhedron*, **18**, 273–279.

[bb3] Brandenburg, K. (1998). *DIAMOND* Crystal Impact GbR, Bonn, Germany.

[bb4] Bruker (2000). *SMART* and *SAINT-Plus* Bruker AXS Inc., Madison, Wisconsin, USA.

[bb5] Hartshorn, C. M. & Steel, P. J. (1998). *J. Chem. Soc. Dalton Trans.* pp. 3935–3940.

[bb6] McMorran, D. A. & Steel, P. J. (2003). *Tetrahedron*, **59**, 3701–3707.

[bb7] Sheldrick, G. M. (2008). *Acta Cryst.* A**64**, 112–122. 10.1107/S0108767307043930 18156677

